# Concordance of patient beliefs and expectations regarding the management of low back pain with guideline recommendations – a cross-sectional study in Germany

**DOI:** 10.1186/s12875-020-01352-1

**Published:** 2020-12-21

**Authors:** Simone Kiel, Christina Raus, Elizabeth Sierocinski, Peggy Knauthe, Jean-François Chenot

**Affiliations:** 1 Department of General Practice, Institute of Community Medicine, University Medicine Greifswald, Fleischmannstraße 6, 17475 Greifswald, Germany; 2 General Practice, Peggy Knauthe, Reinberg, Germany

**Keywords:** Clinical guidelines, Low back pain, Patient expectations, Patient preferences, Non-adherence

## Abstract

**Background:**

Low back pain is a common reason for patients to seek medical care. Physician non-adherence to clinical guidelines has been observed. We investigated the extent to which patient expectations correspond to recommendations of the German national guideline for management of low back pain (G-LBP) and whether patient characteristics, history of LBP and previous treatment experience are associated with expectations.

**Methods:**

A cross-sectional study including patients from 13 general practices was conducted. Data were collected using a questionnaire. Inverse probability weights were used to address non-response bias. Descriptive analysis and multivariate logistic regression models were performed.

**Results:**

A total of 977 patients were included in analyses (median age 57 years, 39% male). 75% of patients reported experiencing LBP currently or within the last year. More than 65% indicated they would agree to forgo further examinations if their LBP was judged by their physician to be of no serious concern. This was associated with the highest level of education and no prior imaging, and negatively associated with good-to-poor health status and moderate-to-severe pain intensity. 40% of participants expected imaging. The highest educational level, female gender and no prior imaging were associated with a decreased expectation of imaging. 70% expected prescriptions for massages. Females, participants with good-to-poor health status, current LBP or LBP in the last 12 months had an increased expectation for massages. Expectations for injection therapy (45%) were mainly associated with previous injections. Expectations for physiotherapy (64%) were associated with female gender, lower educational level, good-to-poor health status, current LBP or in the last 12 months. The perspective that daily activities should be continued (66%) was associated with female gender and higher educational level. Participants who agreed to the statement ‘There is no effective treatment for LBP’ (11%) had a poor health status, current LBP and a severe pain intensity.

**Conclusion:**

Patient views regarding LBP management are partially concordant with guideline recommendations and are strongly influenced by previous treatment experiences and education level. Exploration of patient expectations and experiences in LBP treatment may help minimize dissatisfaction of patients expecting treatments not endorsed by guidelines and simultaneously increase physician guideline adherence.

## Background

Low back pain (LBP) is the global leading cause of years lived with disability (YLDs) [1]. A 17% increase of YLDs attributable to LBP was observed worldwide from 2007 to 2017 [1]. In Germany, 15–20% of the population suffers from chronic LBP [2] and 25% of all adults with statutory health insurance consult a physician for LBP at least once a year [3]. The German national guideline for management of LBP (G-LBP) (updated in 2017 and in line with international guidelines) recommends to refrain from advanced diagnostics such as imaging and laboratory examinations if clinical examination indicates non-specific LBP and to encourage physical activity [4, 5]. Non-specific LBP is defined as the absence of a specific pathology, such as tumour, infection or fracture [6]. LBP related to serious diseases is rare in primary care [4–6].

Variations in diagnostic procedures and treatment of LBP as well as non-adherence to guidelines have been observed in general practice [7–10]. There is evidence that the impact of clinical guidelines for LBP management on clinical practice is minimal [11–13]. Reasons for non-adherence to guidelines by clinicians include lack of awareness of new guidelines, insufficient time and resources to offer recommended care [11, 14, 15]. Additionally, patient preferences and expectations for the management of LBP strongly influence physician adherence to guideline-based recommendations [16]. For example, general practitioners (GPs) may fear that patients will consult another general practice if their expectations are not met [15]. Also, a qualitative study found that GPs were concerned that postponing diagnostic and therapeutic interventions in LBP would cause patients to feel that their pain was not being taken seriously and that their condition was being downplayed [15]. However, fulfilling patient treatment preferences is only inconsistently associated with successful treatment outcomes [17–19]. Patient beliefs, expectations and preferences must be taken into account in order to prevent non-adherence to treatment plans as well as unnecessary use of healthcare resources such as imaging [20–22]. Based on this information, it is possible that the implementation of the G-LBP is influenced by patient expectations and beliefs.

The aim of this study was to investigate the extent to which patient expectations regarding imaging, massages, injection therapy, physiotherapy and maintaining daily activity coincide with the recommendations of the German national disease management guideline for LBP (G-LBP). The secondary aim was to investigate the associations between patient characteristics, patient history of LBP, and previous treatment experiences with beliefs and expectations regarding the management of LBP.

## Methods

### Study design and setting

This cross-sectional study was carried out from June to September 2018 in 13 general practices in Mecklenburg-Vorpommern, Germany. For 3 days, the nurses employed in the practices approached all patients entering the practice, regardless of reason for consultation. This was done to assess whether expectations regarding LBP management differ between individuals with and without current LBP. Practice nurses approached patients consecutively and provided written information. After providing informed consent, patients received a questionnaire to fill out prior to their consultation with the GP. The anonymous questionnaires were placed in a secure container to ensure data privacy. The practice nurse kept a list of all patients with an anonymous, consecutive number, year of birth, gender, participation / non-participation and reason of non-participation (where applicable).

### Exclusion criteria

**Fig. 1:**
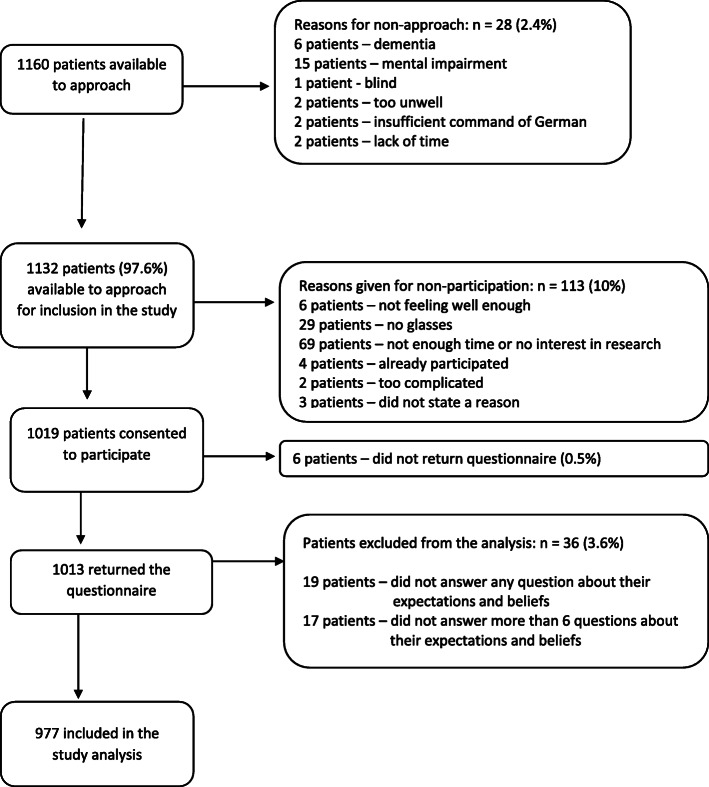
Flow chart of participant recruitment

Patients who were not able to give written informed consent and/or patients under the age of 18 and/or patients with insufficient German language skills were not eligible for the study (Fig. 1, Reasons for non-approach). Eight patients < 18 years were mistakenly approached by the practice nurses. Those eight patients were excluded from the sample because they did not meet the inclusion criteria.

### Questionnaire

The questionnaire was developed based on core recommendations of the German LBP guideline (G-LBP) [23] and the Back Beliefs Questionnaire from Jenkins et al. [24]. Demographic data, current health status and history of LBP were assessed. Patients who reported LBP during the last 12 months were prompted to answer 7 additional questions about pain intensity, impairment of daily activity, and any imaging for LBP during the last 12 months (items 6–12, Additional file 1). Patients who denied having back pain in the last 12 months skipped these questions. Agreement with statements extracted from the core recommendations of the G-LBP regarding diagnosis and treatment of LBP, specifically imaging, massages, injections, physiotherapy, and maintaining daily activity, were measured on a 4-point Likert scale ranging from *strongly agree* to *strongly disagree*. A neutral midpoint was not included, but participants had the option to answer “Don’t know”. Validated questionnaires regarding patient expectations, beliefs and their concordance with recommendations of the national guideline for management of LBP are not available. Instead, the questionnaire was first piloted with 12 patients who volunteered reading the statements aloud and explaining their understanding. Additionally, we piloted the questionnaire in three practices with 139 patients. Based on the feedback, we rephrased several questions, reduced the number and changed the order of the questions. An English translation of the questionnaire is provided in the Additional file 1.

### Data analyses

#### Bias

Out of 1160 consulting patients, 1013 agreed to participate and submitted their responses to the questionnaire (response rate: 87%) (Fig. 1). The Mann-Whitney-U-Test was used to determine age differences and the chi-square test to determine gender differences between participants and non-participants. We found that non-participants (*n* = 147) were older (*p* < 0.0001) and more frequently males (*p* = 0.001) (Table 1). To address this nonresponse bias, inverse probability weights (ipw) were calculated. To calculate ipw, a logistic regression was performed using study participation (yes/no) as the outcome, and age and gender as predictors. The reciprocal of participation probability was considered in the analysis.

**Table 1:** Baseline characteristics of participants and non-participants

	Participants *n* = 1013	Non-participants *n* = 147	*p*-value
Median age in years (Q_1_; Q_3_)	57 ^a^ (41; 68)	66 (52; 80)	< 0.0001
Gender			
Men, n (%)	402 (39,7%) ^b^	79 (53.7%) ^c^	0.001

^a^ missing *n* = 4, ^b^ missing *n* = 3, ^c^ missing *n* = 2

#### Outcomes and predictor variables

Outcome variables were the response categories, dichotomized in agreement (strongly agree and agree) and disagreement (strongly disagree and disagree). Participants who answered ‘Don’t know’ were excluded from the particular regression model. The predictor age was kept as a continuous variable. Educational level was grouped into < 10 years (lower secondary school, other, and no school graduation), 10 years (secondary school, polytechnical institute) and > 10 years (high school, advanced college entrance qualification) of education. Self-assessed health status was grouped in excellent or very good, good, fair, or poor. The predictor ‘low back pain on a 10-point scale’ was grouped into *mild* (0–5 points), *moderate (*6–7 points) and *severe (*8–10 points), corresponding to a study from Boonstra et al. [25]. Prior imaging for LBP (Yes/No) and analgesics for LBP (Yes/No) referred to the last 12 months. Prior injection therapy (Yes/No) referred to ‘ever’ having injection therapy for LBP.

#### Statistical methods

Participants who did not answer more than six questions about their beliefs and expectations (*n* = 36) were excluded from the analysis. Data analysis was of descriptive-explorative nature. Descriptive statistics were used to assess response frequency. Multivariate logistic regression analysis was used to assess the association between the predictor variables age, gender, educational level, self-assessed health status, current LBP or history of LBP, prior injection therapy, LBP on a 10-point scale during the last year and prior imaging for LBP. An additional analysis was carried out including only the subgroup of patients endorsing LBP in the last 12 months. This enabled the additional items answered by these patients to be taken into account. The associations were reported as odds ratios. Multicollinearity of the predictor variables was assessed using Pearson correlation matrix. Predictors with a correlation > 0.4 were excluded. The data was structured in a clustered format reflecting the nesting of patients within the 13 practices. Because patient views and expectations regarding back pain may be influenced by the doctor they consult, the responses of patients within the same cluster (medical practice) may thus have been correlated to one another [26]. Intra-class correlation coefficients (ICCs) were used to quantify the proportion of variation in the outcomes attributed to the cluster effect and thus evaluate the independence of individual data. The ICCs of the outcomes ranged from 0 to 0.05, indicating a very low dependence of patient answers on the medical practice they visited. A sensitivity analysis using multivariate generalised logistic mixed regression models was performed. No meaningful differences in the results were found. As a result, the clusters were ultimately not considered in the analysis. Instead, logistic regression models were performed. The discrimination ability was assessed by the areas under the receiver operating characteristic (ROC) curve. Data analysis was planned a priori to assess differences in response according to age, gender, current or recent LBP and other demographic variables and performed using SAS Institute Inc., Cary, NC, USA, Software 9.4.

## Results

### Physician characteristics

Data were obtained from 13 general practices. Of these, 12 were single practices and one was a group practice. Of the 13 participating GPs, 9 (70%) were women and the mean age was 50 years (range = 38–63 years). Nine of them saw 1000–1500 patients in a three-month interval, two saw 750–999 and two saw fewer than 750. The GPs had been practicing for a median of 12 years (Q_1_ = 3; Q_3_ = 16) at the time of study participation.

### Participant characteristics

A total of 977 participants were included in the analysis (median age 57, Q_1_: 40; Q_3_: 67, 39% male, Table 2, Fig. 1**)**. The number of patients participating from each practice ranged from 31 to 96. More than 70% of the participants reported a good-to-excellent health status. Current LBP was reported by 21% of participants and 55% reported LBP in the last year. Approximately 5% had a history of back surgery and 44% had received injection therapy for LBP in the past (Table 2**).**

**Table 2:** Demographics and low back pain characteristics of study participants

Characteristic	All participants	Participants with current low back pain
Age (median, Q_1;_ Q_3_) ^a^	57 (40; 67) ^a^	58 (48; 68) ^d^
Male (n, %)	381/974 (39.1)	76/211 (36)
Education (n, %)		
< 10 years of school	198/961 (20.6)	55/207 (26.6)
10 years of school	519/961 (54.0)	110/207 (53.1)
> 10 years of school	244/961 (25.4)	42/207 (20.3)
Self-assessed health status, n (%) ^b^		
Excellent	23/954 (3.3)	1/208 (0.3)
Very good	126/954 (17.2)	13/208 (9.3)
Good	527/954 (54.6)	85/208 (44.8)
Fair	234/954 (21.0)	86/208 (35.6)
Poor	44/954 (4.0)	23/208 (10.0)
Low back pain (n, %) ^b^		
Currently experiencing low back pain	211/960 (20.6)	–
Low back pain in the last 12 months	501/960 (54.8)	–
Low back pain not in the last 12 months	248/960 (24.6)	–
Only participants with current low back pain or during the last 12 months (*n* = 712)		
Presenting at the GP for low back pain (n, %) ^b^	96/690 (12.5)	71/203 (33.8)
Low back pain on a 10-point scale in the last 12 months ^b^		
Median (Q_1;_ Q_3_)	5.0 (3; 6) ^e^	5.0 (4; 7) ^f^
• Mild (0–5)	439/687 (64.7)	103/203 (54.5)
• Moderate (6, 7)	162/687 (22.9)	56/203 (26.1)
• Severe (8–10)	86/687 (12.4)	44/203 (19.5)
Analgesics for low back pain in the last 12 months (n, %) ^b^	406/712 (56.7)	157/211 (73.9)
Interference of daily activity on a 10-point scale in the last 12 months (median, Q_1;_ Q_3_)	4.0 (3; 6) ^c^	5.0 (3; 7) ^g^
Imaging for low back pain in the last 12 months (n, %) ^b^	194/705 (25.1)	88/207 (39.6)
Surgery for LBP (n, %) ^b^	61/968 (4.7)	25/210 (9.6)
Injection therapy (n, %) ^b^	472 /961 (43.9)	148/2010 (65.9)

^a^ missing = 4, ^b^ weighted percentage, ^c^ missing = 42, ^d^ missing = 2, ^e^ missing = 25, ^f^ missing = 8, ^g^ missing = 7

*Q*_*1*_ First quartile, *Q*_*3*_ Third quartile

Of the participants with LBP currently or during the last 12 months (76%), 36% were male, median age was 56 (Q_1_: 41, Q_3_: 66), 75% had an educational level of ≤10 years of school, 13% were consulting their GP for current LBP at the time of study participation, 25% had imaging in the last year (x-ray, magnetic resonance imaging, or computer tomography) and 57% had taken analgesics for LBP in the last 12 months. This subgroup reported a median LBP intensity of 5 (Q_1_: 3, Q_3_: 6) and a median impairment of daily activity of 4 (Q_1_: 3, Q_3_: 6) on a 10-point scale. Of those reporting severe pain intensity 88% took analgesics in the last 12 months. 72% with moderate and 45% with mild pain intensity took analgesics in the last 12 months.

### Expectations and beliefs

#### Descriptive statistics

More than 65% of respondents indicated that they would agree or strongly agree to refrain from **further examinations** if their LBP was judged by the doctor to be of no serious concern (due to absence of red flags; addressed in Statement 3–1, G-LBP) **(**Table 3**)**. About 40% expected **imaging** in the workup for acute LBP (Statement 3–6, G-LBP), 70% expected a prescription for **massages** (Statement 5–17, G-LBP), 45% **injection therapy** (Statement 6–27, G-LBP) and 64% a referral for **physiotherapy** (Statement 5–6, G-LBP). About 66% agreed that **continuing everyday activities** as much as possible was beneficial in managing back pain (recommendation 4–5 G-LBP) and 11% believed that there is **no effective treatment** for LBP.

**Table 3:** Patient agreement with core guideline recommendations for management of low back pain, weighted percentages (*n* = 977)

Adaptation of statement based on core recommendations from the national guideline for patients	Strongly agree	Agree	Disagree	Strongly disagree	Do not know
If I have low back pain that is judged to be of no serious concern after medical examination, I am willing to refrain from further examination (n, %) ^a^	333 (36.3)	278 (30.7)	118 (12.5)	55 (4.9)	172 (13.9)
If I have acute low back pain, without weakness or loss of sensation (prickle, numbness) in one leg, I expect imaging (x-ray, CT, MRI) (n, %) ^b^	181 (18.0)	220 (22.2)	231 (26.1)	171 (18.4)	159 (14.0)
If I have low back pain, I expect a prescription for massages (n, %) ^c^	267 (25.6)	394 (44.2)	139 (14.2)	75 (7.6)	89 (7.5)
If I have low back pain, I expect injection therapy (n, %) ^d^	146 (13.2)	300 (31.3)	265 (29.4)	130 (14.1)	121 (10.5)
If I have acute low back pain (less than 6 weeks in duration), I expect a referral for physiotherapy (n, %) ^e^	255 (25.4)	345 (38.1)	146 (16.3)	103 (9.7)	113 (9.5)
If I have low back pain, I should continue my everyday activities as much as possible (n, %) ^f^	227 (22.4)	409 (43.7)	147 (16.4)	72 (7.2)	108 (9.3)
There is no effective treatment for low back pain (n, %) ^g^	39 (3.2)	87 (8.2)	231 (25.2)	273 (30.6)	325 (31.1)

^a^ missing = 21 (1.7%), ^b^ missing = 15 (1.4%), ^c^ missing = 13 (1.0%), ^d^ missing = 15 (1.6%), ^e^ missing = 15 (1.1), ^f^ missing = 14 (1.4%), ^g^ missing = 22 (1.7%)

#### Multivariate analysis

##### Refraining from further examinations

The highest educational level (> 10 years, OR: 2.10, 95% CI: 1.75, 2.53) and LBP in the last 12 months were associated with the expectation that no further examinations should take place (Table 4). Compared to those with very good or excellent health status, participants of good or poor health status were less likely to agree to forego further examinations for the workup of LBP. In the subgroup of participants with LBP in the last 12 months, participants with no prior imaging (OR: 2.41, 95% CI: 2.08, 2.79) agreed to refrain from further examinations, while participants with a moderate or severe pain intensity, a poor health status and participants who took analgesics did not agree to refrain from further examinations if their pain was judged to be of no serious concern by their physician (Table 5).

**Table 4:** Factors associated with expectations and beliefs regarding management of low back pain, including all participants with available data, weights are included

	No further examinations (*n* = 753/977)	Imaging in acute LBP (*n* = 765/977)	Prescription for massages (*n* = 836/977)	Injection therapy (*n* = 788/977)	Referral for physiotherapy in acute LBP (*n* = 808/977)	Continue everyday activity (*n* = 811/977)	There is no effective treatment for LBP (*n* = 600/977)
Independent variable	OR (95% CI)	OR (95% CI)	OR (95% CI)	OR (95% CI)	OR (95% CI)	OR (95% CI)	OR (95% CI)
Gender							
Male	1 (Reference)	1 (Reference)	1 (Reference)	1 (Reference)	1 (Reference)	1 (Reference)	1 (Reference)
Female	1.08 (0.96, 1.23)	**0.69 (0.62, 0.76)**	**1.43 (1.28, 1.59)**	**0.81 (0.73, 0.90)**	**1.22 (1.10, 1.35)**	**2.47 (2.22, 2.76**	0.98 (0.85, 1.15)
Age in years (continuously)	1.00 (0.99, 1.00)	1.01 (1.00, 1.01)	1.00 (0.99, 1.01)	1.01 (1.00, 1.01)	1.00 (0.99, 1.01)	1.03 (1.02, 1.04)	1.01 (1.00, 1.02)
Educational level							
< 10 years of school	1 (Reference)	1 (Reference)	1 (Reference)	1 (Reference)	1 (Reference)	1 (Reference)	1 (Reference)
10 years	1.10 (0.94, 1.28)	1.06 (0.93, 1.21)	1.13 (0.98, 1.31)	**0.74 (0.64, 0.84)**	**1.25 (1.09, 1.43)**	**0.95 (0.82, 1.09**	**0.51 (0.43, 0.61)**
> 10 years	**2.10 (1.75, 2.53)**	**0.74 (0.64, 0.85)**	0.85 (0.73, 1.00)	**0.30 (0.26, 0.35)**	0.94 (0.81, 1.09)	**1.93 (1.63, 2.28)**	**0.31 (0.25, 0.38)**
Self-assessed health status							
Excellent/very good	1 (Reference)	1 (Reference)	1 (Reference)	1 (Reference)	1 (Reference)	1 (Reference)	1 (Reference)
Good	**0.61 (0.51, 0.72)**	**1.35 (1.19, 1.52)**	**1.16 (1.02, 1.32)**	**0.79 (0.70, 0.90)**	**1.72 (1.52, 1.94)**	**0.69 (0.61, 0.80)**	0.96 (0.79, 1.17)
Poor	**0.37 (0.31, 0.45)**	**2.36 (2.04, 2.74)**	**1.18 (1.00, 1.39)**	**0.84 (0.72, 0.97)**	**1.45 (1.24, 1.68)**	**0.41 (0.35, 0.49)**	**2.06 (1.66, 2.56)**
Low back pain							
Not in the last 12 months	1 (Reference)	1 (Reference)	1 (Reference)	1 (Reference)	1 (Reference)	1 (Reference)	1 (Reference)
In the last 12 months	**1.67 (1.45, 1.92)**	**1.47 (1.31, 1.65)**	**1.26 (1.12, 1.42)**	**0.85 (0.76, 0.96)**	**1.29 (1.15, 1.45)**	**1.13 (0.99, 1.28)**	**0.80 (0.67, 0.96)**
Now	1.00 (0.85, 1.18)	0.98 (0.85, 1.14)	**1.64 (1.40, 1.92)**	**0.61 (0.52, 0.70)**	**1.39 (1.20, 1.61)**	**2.36 (1.99, 2.80)**	**1.30 (1.06, 1.59)**
Prior injection therapy	–	–	–		–	–	
No				1 (Reference)			
Yes				**2.14 (1.94, 2.35)**			

*OR* Odds ratio, *95% CI* 95% Confidence interval, *LBP* Low back pain, bold numbers are statistically significant

**Table 5:** Factors associated with expectations and beliefs regarding management of low back pain, including only the subgroup of patients endorsing LBP in the last 12 months, weights are included

	No further examinations (*n* = 538/712)	Imaging in acute LBP (*n* = 561/712)	Prescription for massages (*n* = 618/712)	Injection therapy (*n* = 583/712)	Referral for physiotherapy in acute LBP (*n* = 591/712)	Continue everyday activity (*n* = 601/712)	There is no effective treatment for LBP (*n* = 437/712)
Independent variable	OR (95% CI)	OR (95% CI)	OR (95% CI)	OR (95% CI)	OR (95% CI)	OR (95% CI)	OR (95% CI)
Gender							
Male	1 (Reference)	1 (Reference)	1 (Reference)	1 (Reference)	1 (Reference)	1 (Reference)	1 (Reference)
Female	1.19 (1.02, 1.39)	0.90 (0.79, 1.01)	**1.74 (1.53, 1.98)**	1.08 (0.95, 1.22)	**1. 40 (1.23, 1.59)**	**2.15 (1.89, 2.45)**	1.03 (0.85, 1.23)
Age in years (continuously)	1.00 (0.99, 1.01)	1.01 (1.00, 1.01)	1.00 (0.99, 1.01)	1.01 (1.00, 1.02)	1.00 (0.99, 1.01)	1.03 (1.03, 1.04)	1.01 (1.01, 1.02)
Educational level							
< 10 years of school	1 (Reference)	1 (Reference)	1 (Reference)	1 (Reference)	1 (Reference)	1 (Reference)	1 (Reference)
10 years	0.81 (0.65, 0.99)	**0.82 (0.69, 0.96)**	1.06 (0.89, 1.28)	**0.56 (0.48 0.66)**	1.20 (1.02, 1.42)	**0.85 (0.71, 1.01)**	**0.55 (0.45, 0.68)**
> 10 years	1.22 (0.96, 1.56)	**0.55 (0.47, 0.66**)	**0.74 (0.60, 0.90)**	**0.22 (0.19, 0.27)**	1.11 (0.92, 1.33)	**1.34 (1.09, 1.64)**	**0.36 (0.28, 0.47)**
Self-assessed health status							
Excellent/very good	1 (Reference)	1 (Reference)	1 (Reference)	1 (Reference)	1 (Reference)	1 (Reference)	1 (Reference)
Good	0.82 (0.65, 1.03)	**1.77 (1.51, 2.07)**	**1.56 (1.33, 1.84)**	0.97 (0.84, 1.14)	**1.98 (1.70, 2.31)**	**0.63 (0.53, 0.75)**	1.22 (0.94, 1.58)
Poor	**0.66 (0.51, 0.85)**	**2.49 (2.07, 2.99)**	**2.15 (1.75, 2.63)**	0.90 (0.75, 1.09)	**1.78(1.48, 2.15)**	**0.44 (0.36, 0.54)**	**3.21 (2.42, 4.26)**
Low back pain on a 10-point scale in the last year							
Mild (0–5)	1 (Reference)	1 (Reference)	1 (Reference)	1 (Reference)	1 (Reference)	1 (Reference)	1 (Reference)
Moderate (6, 7)	**0.73 (0.62, 0.86)**	1.07 (0.94, 1.22)	0.87 (0.75, 1.00)	**1.23 (1.08, 1.41)**	0.90 (0.78, 1.04)	0.84 (0.73, 0.97)	**0.65 (0.53, 0.80**)
Severe (8–10)	**0.40 (0.33, 0.49)**	1.02 (0.86, 1.22)	1.08 (0.88, 1.32)	0.84 (0.71, 1.00)	0.85 (0.71, 1.03)	**0.56 (0.47 0.68)**	**1.28 (1.02, 1.62)**
analgesics for low back pain in the last 12 months							
No	1 (Reference)	1 (Reference)	1 (Reference)	1 (Reference)	1 (Reference)	1 (Reference)	1 (Reference)
Yes	**0.65 (0.55, 0.76**	**0.85 (0.76, 0.96)**	**0.74 (0.65, 0.85)**	**1.37 (1.22, 1.54)**	**1.25 (1.11, 1.41**	**1.33 (1.16, 1.51)**	0.96 (0.81, 1.15)
Prior imaging for LBP			–	–	–	–	–
Yes	1 (Reference)	1 (Reference)					
No	**2. 41 (2.08, 2.79)**	**0.63 (0.55, 0.71)**					
Prior injection therapy	–	–	–		–	–	
No				1 (Reference)			
Yes				**2.19 (1.96, 2.45)**			

*OR* Odds ratio, *95% CI* 95% Confidence interval, *LBP* Low back pain, bold numbers are statistically significant

##### Imaging

The highest level of education (> 10 years) and female gender were associated with a decreased expectation of imaging, while a good (OR: 1.35, 95% CI: 1.19, 1.52) or -poor (OR: 2.36, 95% CI: 2.04, 2.74) health status and LBP in the last 12 months were associated with an increased expectation of imaging (Table 4). Additionally, in this subgroup, a lack of prior imaging was associated with a decreased expectation of imaging in the workup of LBP (Table 5).

##### Massages

Female participants, participants with good-to-poor health status, and participants with current LBP or LBP in the last 12 months were more likely to expect a prescription for massages (Table 4). In the subgroup analysis, participants with a good-to-poor health status were more likely to expect a prescription for massages. Participants who took analgesics for LBP in the last 12 months did not expect massages (Table 5).

##### Injection therapy

Female participants, participants with a higher educational level (10 years and > 10 years of school) with a good-to-poor health status, and participants with current LBP or LBP in the last 12 months were less likely to expect injection therapy. Participants who had previously received injection therapy in the treatment of LBP had a high expectation of injection therapy (OR: 2.14, 95% CI: 1.94, 2.35) (Table 4). Participants with moderate LBP intensity and participants who took analgesics in the last 12 months were more likely to expect injection therapy (Table 5).

##### Physiotherapy

Female participants, participants with 10 years of education, with good or poor health status, and participants with current LBP or LBP in the last 12 months expected a referral for physiotherapy (Table 4). In participants with LBP in the last 12 months, only the female gender and a good or poor health status and analgesics in the last 12 months were associated with the expectation of physiotherapy (Table 5).

##### Continuing everyday activities

Female participants (OR: 2.47, 95% CI: 2.22, 2.76), participants with a higher educational level (10 years and > 10 years), and participants with current LBP (OR: 2.36, 95% CI: 1.99, 2.80) or LBP in the last 12 months indicated agreement regarding the benefits of continuing everyday activity despite LBP. Participants with good or poor health status (Table 4) and those with severe pain intensity during the last 12 months were more likely to disagree that they should continue everyday activity while experiencing LBP (Table 5). Participants who took analgesics were more likely to agree (Table 5).

##### Belief in effective treatment

Participants with a higher educational level (10 and > 10 years) and participants with LBP in the last 12 months were less likely to agree that there is no effective treatment for LBP. Participants with poor health status (OR: 2.06, 95% CI: 1.66, 2.56) and participants with current LBP were more likely to agree that there is no effective treatment (Table 4). Participants experiencing moderate-intensity LBP were less likely to believe that there is no effective treatment for LBP while participants with severe LBP were more likely to believe there is no effective treatment (Table 5).

## Discussion

### Summary of main results

More than half of participants (67%) indicated that they would agree to refrain from further examinations if their LBP was judged by their doctor to be of no serious concern (absence of red flags). About 40% expected imaging workup, 70% a prescription for massages, 45% injection therapy, and 64% a referral for physiotherapy. About 66% agreed to continue everyday activity despite LBP and 11% believed there is no effective treatment for LBP. These expectations and beliefs were dependent on gender, educational level, self-assed health status, current LBP and LBP in the last 12 months, pain intensity, use of analgesics, prior imaging and prior injection therapy.

### Interpretation of results

#### Suffering from and seeking care for LBP

Our study findings correspond to the one-year LBP prevalence of 76% in the German adult population reported in another study [27]. The majority of patients with a history of LBP in the last 12 months were female. Gender imbalances in LBP are also shown in other studies [28–30]. However, in our study the majority of people suffering from LBP were not actively seeking medical care at the time of participation. Only 13% of participants with current LBP or LBP during the last 12 months listed LBP as the reason for GP consultation on the questionnaire. When considering only participants with current LBP the percentage increased to 34%. It is possible that these participants had already sought care for their LBP or were planning to in the future. In Germany, roughly 25% of the population annually seek medical care for LBP [3]. The majority of participants suffering from LBP during the last 12 months had a lower educational level (≤ 10 years). This result is concordant with a German study [31], where 83% of participants with lower educational levels suffered from LBP compared to 62% of those with university degrees.

#### Refraining from further examinations

The agreement from 67% of participants to refrain from further examinations in the absence of “red flags” is in accordance with national and international guidelines for the management of LBP. Of note, participants who indicated agreement to forego further testing had no prior imaging for LBP. Participants suffering from moderate-to-severe LBP currently or within the last 12 months did not agree to refrain from further examinations. Similar results were found in a qualitative Australian study, where patients with severe pain were more likely to expect x-rays and prior experiences of care had an influence on current expectations regarding LBP management [32]. Also, participants who took analgesics in the last 12 months did not agree to refrain from further examinations. These results are in line with the pain intensity. Although, not all participants with severe pain took analgesics in the last 12 months. *Expectations for imaging.* A report including more than 5 million German patients covered by statutory health insurance with the billing diagnosis of back pain found that 375 of 1000 patients had imaging workup during 1 year [33]. The imaging rate in our study population was lower (275 per 1000) but still suggests overuse given that imaging is only recommended with high suspicion for specific LBP (Statement 3–6, G-LBP). The high frequency of imaging in LBP patients and the lack of clinical benefit is a well-known phenomenon [33–35]. A systematic review and meta-analysis concluded that abnormalities identified by MRI are very common even in asymptomatic individuals and do not coincide with LBP development [36]. Imaging workup in patients with non-specific LBP has also been shown to be of no clinical benefit for the patient [14, 37]. Moreover, imaging workup in LBP can lead to increased surgery rates [14, 33, 37]. In our study, 40% of patients believed imaging was necessary in the workup and treatment of LBP. In the entire study population, the highest educational level (> 10 years of school) and female gender were associated with a decreased expectation for imaging. In patients with LBP currently or within the last year, a lack of prior imaging was associated with a decreased expectation of imaging. A patient survey from Jenkins et al. [24] found a similar rate (48%) of patients expecting imaging, with the same associations. The association between previous imaging and the strong belief in the need for imaging seems to be the basis for a cycle of health care use in LBP [24]. It is unknown if this patient belief is influenced by the treating clinicians [24]. A systematic review of interventions to reduce imaging in LBP showed inconsistent effects [35]. To our knowledge, there exist no studies assessing the best way for physicians to communicate the limited benefit of imaging in LBP to patients.

#### Expectations for injection therapy

The G-LBP firmly recommends against the injection of non-steroidal anti-inflammatory drugs (NSAIDs). Injections are not superior to oral applications and have additional injection-related risks of complications such as infection or physical injury [5, 38]. However, GPs assume that patients insist on injection therapy because they believe intramuscular injections lead to a faster and better pain relieve compared to oral application [38]. A large proportion (45%) of participants in our study expected injection therapy in the treatment of LBP. These participants often had previously received injection therapy for LBP (OR: > 2) and took analgesics for LBP during the last 12 months. The previous experience of analgesics and injection therapy may imply a similar cycle of reinforcement as seen with previous imaging. Increasing patient education and awareness may help to interrupt this cycle. It has been shown that providing patients with information about the risks of oral versus injected NSAID influences patients decisions regarding mode of administration [38]. According to our findings, patients with LBP and no previous injection therapy do not expect injection therapy.

#### Massages and physiotherapy

The G-LBP does not recommend massages for LBP and physiotherapy is only recommended for subacute (lasting 4–12 weeks) and chronic (lasting > 12 weeks) LBP due to insufficient evidence of effective treatment [4, 5]. Yet, most patients (70%) expected massages and 63% expected physiotherapy. This expectation was mainly associated with female gender and a good-to-poor health status. Participants with current LBP had an increased expectation of massages and referral to physiotherapy.

#### Continuing everyday activities

Two-thirds (66%) of patients agreed to continue their everyday activities despite LBP. In a cohort study from Werber et al. [31], only 38% considered maintaining physical activities as helpful while experiencing LBP. We observed that a higher educational level (≥ 10 years) and female gender were associated with the belief that everyday activity should be continued, while participants with a good-to-poor health status disagreed. Overall, patients with current LBP were more likely to agree that everyday activities should be continued, potentially reflecting recent physician recommendations to continue daily activities. However, of patients with current or recent LBP, those with severe pain disagreed that everyday activity should be continued. This finding is in accordance with the literature, which shows that patients reporting higher levels of disability more strongly believed they should avoid physical activity (also known as fear of pain due to movement or kinesiophobia) [22, 39, 40].

#### Belief in effective treatment

Participants with a higher educational level (≥ 10 years) and LBP in the last year were less likely to believe there is no effective treatment for LBP while participants with a poor health status, current LBP, and severe pain intensity believed there is no effective treatment. Werber et al. also found significant differences in educational levels of patients endorsing varying perspectives on LBP management [31].

#### Strategies to influence patient expectations

The influence of public campaigns to change patient beliefs about back pain was investigated in four large studies (in Australia, Norway, Canada, and Scotland). The public health campaign in Australia was successful in altering general population beliefs and beliefs about back pain [41]. The mass media campaigns in Canada and Norway only had a small impact on altering back pain beliefs [42, 43] and the campaign in Scotland lead to a major shift in public beliefs but exerted no impact on work-related outcomes [44]. It was discussed that the small impact of the Canadian and Norwegian campaign may be due to the relatively small target area, low budget, and failure to directly address work-related issues [42–44]. Policymakers and third-party payers assessing guideline adherence using quality indicators should consider the difficult position of clinicians as they seek to balance patient expectations and preferences with potentially conflicting guideline recommendations.

We found that prior treatment experience exerted a strong influence on patient expectations (OR > 2) compared to other factors with mostly small influences on expectations. As a result, a feasible strategy to align LBP treatment in practice with guidelines could be to avoid initiation of non-recommended therapies in patients seeking care for LBP for the first time. This can potentially change patient expectations of future management of LBP and decrease pressure on physicians.

### Strengths and limitations

Considerable strengths of this study are the large sample and the high response rate of 87%. Inverse probability weights were used to account for the nonresponse bias [45]. Furthermore, the results remained robust in a sensitivity analysis considering the clustered structure, confirming the independence of individual data. Our questionnaire was tailored to the G-LBP; however, for this reason we could not use a validated instrument. Our questionnaire was piloted in three general practices with 139 patients. Additionally, cognitive interviewing was used on a subsample as a method of validity evidence [46]. Patients completed the questionnaire before seeing their GP, a measure meant to minimize potential GP-influence on patient answers. In addition, patients were asked to recall their history of LBP, which could have caused a recall bias. However, the influence from a recall bias may be low, because we limited the recall time to the last 12 months. We did not collect data on income, working status and specific co-morbidities, which might have affected responses. However, these factors are partially reflected by educational status, health status, and being of working age. We may underestimate patient disagreement with guideline recommendations due to social desirability bias [47]. Patient expectations are shaped by the health care system, e.g. access to care or co-payments; this should be kept in mind when generalizing our data.

### Conclusion

Our study indicates that patient views regarding management of LBP in an ambulatory care setting are only partially concordant with guideline recommendations for LBP. Patient beliefs and expectations regarding LBP management are strongly influenced by previous treatment experiences, educational level, and, in some cases, gender. Given the potential impact on patient satisfaction, further exploration of previous treatments experienced by patients and their current expectations may help increase physician guideline adherence and minimize the dissatisfaction of patients who expect treatments not endorsed by guidelines.

## Supplementary Information

**Additional file 1.**

## Data Availability

The dataset used and analysed during the current study are available from the corresponding author on reasonable request.

## Abbreviations

G-LBP: Guideline for management of low back pain
GP: General Practitioner
ICC: Intraclass correlation coefficient
ipw: Inverse probability weight
LBP: Low back pain
NSAIDS: Nonsteroidal anti-inflammatory drugs
Q_1_: First quartile
Q_3_: Third quartile
YLD: Years lived with disability
