# Supplementary material for: Concordance of patient beliefs and expectations regarding the management of low back pain with guideline recommendations – a cross-sectional study in Germany
Source: BMC Fam Pract. 2020 Dec 21;21:275. doi: 10.1186/s12875-020-01352-1 (PMC7751122; doi:10.1186/s12875-020-01352-1)
Supplement: Supplementary file 1 — Additional file 1. [file 12875_2020_1352_MOESM1_ESM.docx]

| number of practice | | | | | | | | | | |  | | 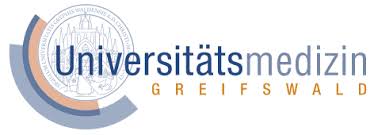 | | | | |
| --- | --- | --- | --- | --- | --- | --- | --- | --- | --- | --- | --- | --- | --- | --- | --- | --- | --- |
|  |  |  |  |  |  |  |  |  |  | |  | | | | | | |
|  | | | | | | | | | | |  | | | | | | |
| consecutive number | | | | | | | | | | |  |  | |  |  |  | |
|  | | | | | | | | | | |  | |  | | |  | |
| **Survey on Low Back Pain (Translation)** | | | | | | | | | | | | | | | | | |
|  | | | | | | | | | | |  | |  | | | |  |
|  | | | | | | | | | | |  | | **Participant Questionnaire** | | | |  |
|  | | | | | | | | | | | | | | | | | |
| Please complete all pages of this questionnaire and use a pen or fine liner. Answer each question and use the option “Cannot answer” if needed. You are asked to skip questions that do not apply to you. This is indicated with the note: “Please proceed with question 13”. If you have accidentally ticked an incorrect answer, please blacken the wrong selection and mark the correct box. | | | | | | | | | | | | | | | | | |
| mark as follow: | | | | | | | | | | 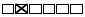 | | | | | | | |
| correct as follow: | | | | | | | | | | 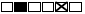 | | | | | | | |
|  | | | | | | | | | |  | | | | | | | |
|  | | | | | | | | | | | | | | | | | |
| blinded manuscript | | | | | | | | | | | | | | | | | |
|  | | | | | | | | | | | | | | | | | |
|  | | | | | | | | | | | | | | | | | |

| 1 | | | Year of Birth: | | | _ _ _ _ (year) | | | | | | | | | | | | | | | |  | | | | | | | | | | | | |
| --- | --- | --- | --- | --- | --- | --- | --- | --- | --- | --- | --- | --- | --- | --- | --- | --- | --- | --- | --- | --- | --- | --- | --- | --- | --- | --- | --- | --- | --- | --- | --- | --- | --- | --- |
|  | | | | | | | | | | | | | | | | | | | | | | | | | | | | | | | | | | |
| 2 | | | Gender: | | Male 🞎 | | | | | | Female 🞎 | | | | | | | | | | |  | | | | | | | | | | | | |
|  | | | | | | | | | | | | | | | | | | | | | | | | | | | | | | | | | | |
| 3 | | What is the highest level of education  that you have completed? | | | | | | | Lower secondary school | | | | | | | | | | | | | | | | | | | | | 🞎 | | | | |
|  |  |  |  |  |  |  |  |  | Secondary school | | | | | | | | | | | | | | | | | | | | | 🞎 | | | | |
|  |  |  |  |  |  |  |  |  | Polytechnical institute | | | | | | | | | | | | | | | | | | | | | 🞎 | | | | |
|  |  |  |  |  |  |  |  |  | Advanced college entrance qualification | | | | | | | | | | | | | | | | | | | | | 🞎 | | | | |
|  |  |  |  |  |  |  |  |  | High School | | | | | | | | | | | | | | | | | | | | | 🞎 | | | | |
|  |  |  |  |  |  |  |  |  | Other school graduation | | | | | | | | | | | | | | | | | | | | | 🞎 | | | | |
|  |  |  |  |  |  |  |  |  | No school graduation | | | | | | | | | | | | | | | | | | | | | 🞎 | | | | |
|  | | | | | | | | | | | | | | | | | | | | | | | | | | | | | | | | | | |
| 4 | | | How would you rate your current health status? | | | | | | | | | | | | | | | | | | | | | | | | | | | | | | | |
|  | | | excellent 🞎 | very good 🞎 | | | | good 🞎 | | | | | | | fair 🞎 | | | | | | | | | poor 🞎 | | | | | | | | | |  |
|  | | | | | | | | | | | | | | | | | | | | | | | | | | | | | | | | | | |
| 5 | | | Have you experienced low back pain?  (pain anywhere in the area between the lowest rib and the buttock crease, as pictured) | | | | 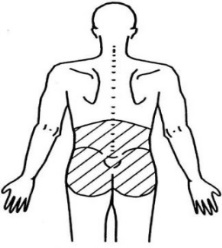 | | | | | | | | | Now | | | | | | | | | | | | | | | | 🞎 | | |
|  |  |  |  |  |  |  |  |  |  |  |  |  |  |  |  | In the last 12 months | | | | | | | | | | | | | | | | 🞎 | | |
|  |  |  |  |  |  |  |  |  |  |  |  |  |  |  |  | I have not experienced low back pain within the last 12 months: ⇒ please proceed with question 13 | | | | | | | | | | | | | | | | 🞎 | | |
|  | | | | | | | | | | | | | | | | | | | | | | | | | | | | | | | | | | |
| 6 | | | Are you presenting to your General Practitioner for low back pain today? | | | | | | | | | | | | | | | No 🞎 | | | | | | | | Yes 🞎 | | | | | | | | |
|  | | | | | | | | | | | | | | | | | | | | | | | | | | | | | | | | | | |
|  |  | | | | | | | | | 0=No Pain 10=Pain as bad as it could be | | | | | | | | | | | | | | | | | | | | | | | | |
| 7 | | How would you rate your low back pain on a 0-10 scale **at the present time**? | | | | | | | | 0  🞎 | | 1  🞎 | 2  🞎 | | | 3  🞎 | 4  🞎 | | | 5  🞎 | | | 6  🞎 | | 7  🞎 | | | 8  🞎 | | | 9  🞎 | | 10  🞎 | |
| 8 | | How intense was your low back pain on average on a 0-10 scale **in the last 12 months**? | | | | | | | | 0  🞎 | | 1  🞎 | 2  🞎 | | | 3  🞎 | 4  🞎 | | | 5  🞎 | | | 6  🞎 | | 7  🞎 | | | 8  🞎 | | | 9  🞎 | | 10  🞎 | |
|  | | | | | | | | | | | | | | | | | | | | | | | | | | | | | | | | | | |
|  | | | | | | | | | | | | | | | | | | No | | | | | | | | | | Yes | | | | | | |
| 9 | Have you taken analgesics for low back pain in the last 12 months? | | | | | | | | | | | | | | | | | 🞎 | | | | | | | | | | 🞎 | | | | | | |
|  | | | | | | | | | | | | | | | | | | | | | | | | | | | | | | | | | | |
|  |  | | | | | | | | | 0=No pain 10=Pain as bad as it could be | | | | | | | | | | | | | | | | | | | | | | | | |
| 10 | How much does low back pain currently interfere with your daily activity? | | | | | | | | | 0  🞎 | | 1  🞎 | 2  🞎 | | | 3  🞎 | 4  🞎 | | | 5  🞎 | | | 6  🞎 | | 7  🞎 | | | 8  🞎 | | | 9  🞎 | | 10  🞎 | |
| 11 | In the last 12 months, how much has low back pain interfered with your daily activities? | | | | | | | | | 0  🞎 | | 1  🞎 | 2  🞎 | | | 3  🞎 | 4  🞎 | | | 5  🞎 | | | 6  🞎 | | 7  🞎 | | | 8  🞎 | | | 9  🞎 | | 10  🞎 | |
|  | | | | | | | | | | | | | | | | | | | | | | | | | | | | | | | | | | |
| 12 | Which diagnostic imaging for low back pain have you had in the last 12 months? (multiple answers possible) | | | | | | | | | x-ray | | | | MRI | | | | | CT | | | | | | | | None | | | | | | | |
|  |  |  |  |  |  |  |  |  |  | 🞎 | | | | 🞎 | | | | | 🞎 | | | | | | | | 🞎 | | | | | | | |
|  | | | | | | | | | | | | | | | | | | | | | | | | | | | | | | | | | | |
| 13 | Have you ever had surgery for low back pain? | | | | | | | | | | | | | | | | | | | | No 🞎 | | | | | | | | Yes 🞎 | | | | | |
| 14 | Have you ever had injection therapy for low back pain? | | | | | | | | | | | | | | | | | | | | No 🞎 | | | | | | | | Yes 🞎 | | | | | |

|  |  | strongly agree | agree | disagree | strongly disagree | Do not know |
| --- | --- | --- | --- | --- | --- | --- |
| 15 | If I have low back pain that is judged by the doctor to be of no serious concern after medical examination, I am willing to refrain from further examinations. | 🞎 | 🞎 | 🞎 | 🞎 | 🞎 |
| 16 | If I have acute low back pain, without weakness or loss of sensation (prickle, numbness) in one leg, I expect imaging (x-ray, CT, MRI). | 🞎 | 🞎 | 🞎 | 🞎 | 🞎 |
| 17 | If I have low back pain, psychological problems may also play a role. | 🞎 | 🞎 | 🞎 | 🞎 | 🞎 |
| 18 | If I have low back pain, I expect the doctor to ask questions about stress situations in my family/at my workplace and depressive symptoms/anxiety. | 🞎 | 🞎 | 🞎 | 🞎 | 🞎 |
| 19 | If I have low back pain, a psychologist / psychotherapist can help identify accompanying mental health problems. | 🞎 | 🞎 | 🞎 | 🞎 | 🞎 |
| 20 | If I have low back pain, I expect a prescription for massages. | 🞎 | 🞎 | 🞎 | 🞎 | 🞎 |
| 21 | If I have chronic low back pain, co-treatment by a psychologist/psychotherapist can help. | 🞎 | 🞎 | 🞎 | 🞎 | 🞎 |
| 22 | If I have low back pain, I expect injection therapy. | 🞎 | 🞎 | 🞎 | 🞎 | 🞎 |
| 23 | If I have low back pain, I should continue everyday activities as much as possible. | 🞎 | 🞎 | 🞎 | 🞎 | 🞎 |
| 24 | If I have acute low back pain (less than 6 weeks in duration), I expect a referral for physiotherapy. | 🞎 | 🞎 | 🞎 | 🞎 | 🞎 |
| 25 | Regular exercise is important to relieve my pain. | 🞎 | 🞎 | 🞎 | 🞎 | 🞎 |
| 26 | If I have low back pain, learning relaxation techniques is a good way to relieve my pain. | 🞎 | 🞎 | 🞎 | 🞎 | 🞎 |
| 27 | If I have low back pain, I must rest. | 🞎 | 🞎 | 🞎 | 🞎 | 🞎 |
| 28 | There is no effective treatment for low back pain. | 🞎 | 🞎 | 🞎 | 🞎 | 🞎 |
| 29 | Doctors cannot do anything for low back pain. | 🞎 | 🞎 | 🞎 | 🞎 | 🞎 |
| **Thank you for completing this questionnaire.** | | | | | | |
